# Neurofibromatosis type 1 and multiple traumatic cervical arterial injuries: a case report

**DOI:** 10.4076/1757-1626-2-7199

**Published:** 2009-08-07

**Authors:** Bertrand Sauneuf, Stéphanie Chevalier, Claude Jehan, Patrick Courtheoux, Jean-Louis Gérard, Jean-Luc Hanouz, Benoit Plaud

**Affiliations:** 1 Pôle d’Anesthésie Réanimation chirurgicale SAMU-SMURCHU, Avenue de la Côte de Nacre, 14033 CaenFrance; 2 Pôle d’Imagerie MédicaleCHU, Avenue de la Côte de Nacre, 14033 CaenFrance; 3 Université de Caen Basse-Normandie, France

## Abstract

**Introduction:**

Blunt injury to the carotid and vertebral arteries is uncommon and potentially devastating. Neurofibromatosis type 1, or von Recklinghausen’s disease is an autosomal dominant disorder affecting one in 3000 individuals. This genetic disease may affect many organs, including vessels.

**Case presentation:**

This report describes a very unusual case of multiple traumatic arterial injuries in a 44-year-old Caucasian neurofibromatosis type 1 patient, with delayed diagnosis. The vascular abnormalities observed in neurofibromatosis type 1, probably enhanced the arterial lesions. Medical treatment with antiplatelet therapy combined with endovascular stent-assisted angioplasty allowed a good evolution for the patient.

**Conclusion:**

Patients with medical history of neurofibromatosis type I are at risk to develop blunt cervical vascular injury. This association has to be known by emergency and intensivist physicians to optimize screening of cerebrovascular injuries and treat early such devastating lesions.

## Introduction

Among patients admitted to the hospital for blunt trauma, the incidence of carotid artery injury is approximately 1% [1]. Arterial dissection occurs when an intimal tear allows blood to enter the arterial wall, potentially compromising the lumen and reducing blood flow. Thrombosis and cerebral ischemia can result. Arterial dissection can also arise spontaneously [2] and has been associated with hypertension, drug abuse, fibromuscular dysplasia, Ehlers-Danlos and Marfan syndrome or neurofibromatosis type (NF-1) [3]. Vertebral arteriovenous fistulae are less common complications, which may occur following blunt trauma associated with other cervical artery lesions. There have been reports of spontaneous vertebral arteriovenous fistulae associated with NF-1 [4].

Neurofibromatosis type 1 or von Recklinghausen’s disease is one of the most common genetic disease affecting 1/3000 to 1/4000 individuals. It has a wide variability of clinical expression affecting skin, eyes, bones, nervous and vascular system [5]. The most common vascular abnormality in patients with NF-1 is renal artery stenosis. When cerebrovascular abnormalities occur, these are usually occlusion and stenosis of major intracranial vessels. Aneurysms and arteriovenous malformations affect 5% of those patients [6].

## Case presentation

A 44-year-old Caucasian male had a road traffic accident. The patient had a medical history of NF-1 (von Recklinghausen’s disease). On arrival in the trauma center, peripheral blood pressure was 145/80 mmHg, heart rate 130 beats/min, peripheral oxygen saturation 97% with oxygen at 10 L/min, initial Glasgow coma scale scored 14. Except for disorientation, the patient showed no neurologic abnormalities. He complained of chest pain and headache. Physical examination was typical for Recklinghausen’s disease with *café-au-lait* spots and neurofibromas on his trunk and limbs. The patient had areas of bruising on the chest in a seat belt distribution pattern. Initial capillary haemoglobin was 9.7 g/dL (HemoCue B-Hemoglobin®, HemoCue AB™, Ängelholm, Sweden). A total of 1000 mL sodium chloride 0.9 % and 500 ml Gelofusin® (B.Braun AG™, Melsungen, Germany) was infused as fluid resuscitation. For pain control 100mg tramadol and 1g paracetamol were injected. Patient underwent bilateral thoracic drainage because of signs of bilateral pneumothorax on chest X-ray.

**Figure 1.**
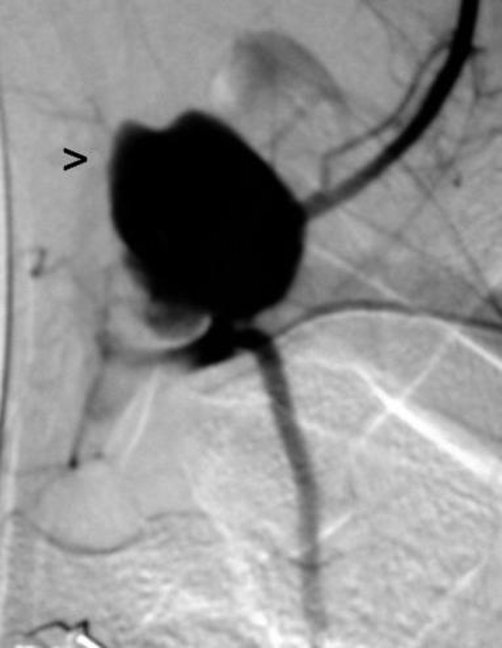
Angiogram of the left subclavian artery and his branches. Vascular lake due to extravasation of contrast product (black arrow).

A cerebral computerized tomography scan showed right sphenoïdal hemosinus. An angio CT scan detected traumatic subarachnoïdal hemorrhage, without intracranial vascular abnormality. A thorax CT scan revealed a bilateral anterior pneumothorax, a left hemothorax and a left subclavian artery branch lesion, with active bleeding. One hour after the first haemoglobin determination, the concentration decreased to 6.2 g/dL. A total of seven units of packed red blood cells and three units of fresh frozen plasma were administered in the six hours after admission. The patient was anesthetized, intubated (rapid sequence induction with etomidate and succinylcholine) and mechanically ventilated to prevent breathing difficulties due to a progressively expanding hematoma in the left supraclavicular and mediastinum area. An angiography of the aortic arch, that didn’t include the neck, revealed active bleeding on a collateral artery of the thyrocervical trunk, a branch from the left subclavian artery (Figure 1), and was treated by intravascular covered stent. The patient remained hemodynamically stable and was admitted in intensive care.

**Figure 2.**
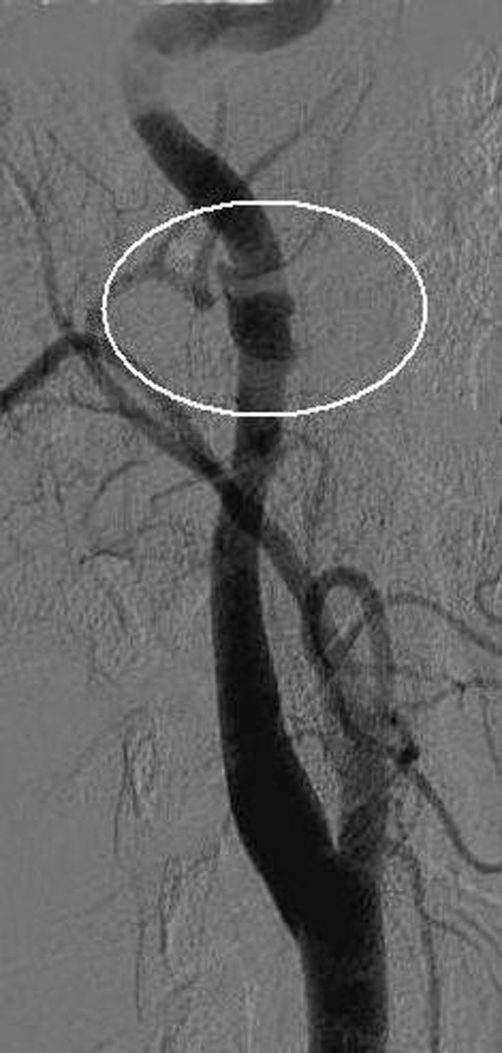
Angiogram of the right carotid artery. Traumatic pseudo aneurysm (white circle)

**Figure 3.**
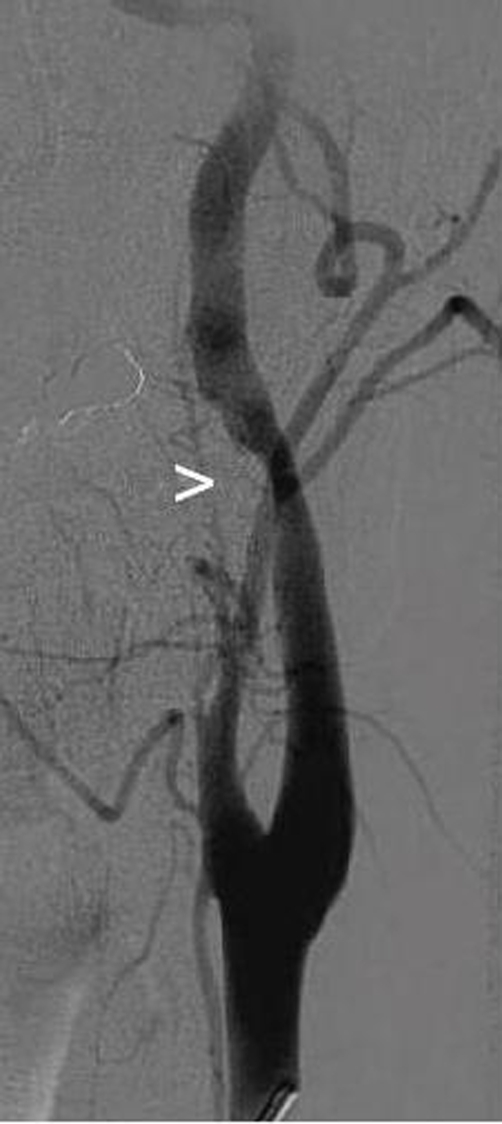
Angiogram of the left carotid artery. Acute stenosis due to dissection (white arrow).

On day 6, the patient was extubated. His neurological examination remained stable. He complained of a constant bruit synchronized with the arterial pulse that he could hear in his head. On day 7, physical examination found a right carotid murmur. A doppler ultrasound found a distal right internal carotid pseudo aneurysm. A cerebral angiography confirmed a right internal carotid pseudo aneurysm (Figure 2), and revealed a left internal carotid artery dissection (Figure 3) and a right vertebral arterio-venous fistula. No sign of fibromuscular dysplasia was found. The patient was first treated with full-dose of standard heparin sodium. On day 8, the right carotid artery underwent balloon angioplasty and stenting. The right vertebral artery was occluded by coils. Heparinization was discontinued 24 hours post-treatment and the patient received oral antiplatelet therapy (clopidogrel bisulfate, one per day).

The patient was discharged from the hospital on day 35, without any neurological sequelae.

## Discussion

Intrinsic lesions of the arterial wall are important manifestations of NF-1, yet the pathogenesis of these lesions remains ill defined. Since Green in 1974, it is known that neurofibromas are associated with degenerative changes of the adjacent wall of large vessels [7]. These pathological changes may cause fragility of the vessel and sometimes result in the formation of aneurysm and/or rupture. On 31 patients presenting 76 vascular abnormalities, Oderich *et al.* found 3 cases of subclavian arteries aneurysms [8]. Seow *et al.* reported a case of left subclavian artery aneurysm rupture leading to hemothorax and airway obstruction [9]. But the usual NF-1 vascular lesions also involve the walls of arteries that are not directly associated with neurofibromas. The pathologic mechanism of arterial lesions may result from obstructive lesions due to intimal thickening. In a recent case of spontaneous rupture of thyrocervical trunk branch, Ishizu *et al.* carried out histological tissue examination and found dysplasia of the smooth muscle layer in the intima and media that probably leads to fragility of the vessel [10]. This pathological finding was limited to the vessel wall without formation of a macroscopic tumor, suggesting that in NF-1 any vessel could be affected.

In the present case, the patient did not have medical history other than NF-1. The multiple localizations made us hypothesize, that the post-traumatic arterial lesions were enhanced by the vascular abnormalities observed in NF-1.

Although unconfirmed in this trauma case, a pre-existing aneurysm of the thyrocervical trunk is suspected. The patient presented with a typical sign of vertebral fistula (i.e. hearing the fistula murmur in his head). By completing the anamnesis, no signs of a fistula were present before the trauma, suggesting a traumatic origin. However, a pre-existing fistula worsened by the trauma or an opening of fistula caused by the trauma, cannot be excluded. The bilateral carotid artery dissection is unusual in NF-1. We exclude that the left carotid dissection could have occurred as a complication from the first procedure that did not explore the neck area. Moreover we were unaware of any reports showing increased risk of complications with angiography in NF-1 patients. The vascular fragility associated with the trauma could explain the clinical findings of our patient.

Screening criteria widely used for suspected blunt cerebrovascular injury, are cervical spine and complex facial fractures, Horner syndrome or anisocoria, unexplained neurologic deficit and high clinical suspicion including neck soft tissue injury. Those criteria should include pre-existing vascular pathology, as NF-1.

The treatment of such injuries remains controversial. Ours included intravascular stenting and antiplatelet therapy, as previously described [11]. The immediate angiographic result and short-term outcome were favourable.

## Conclusion

In summary, any trauma patient with NF-1 should be considered to have a systemic vasculopathy until proven otherwise. In blunt trauma, the fragility of the vessels increases the probability of cerebrovascular lesions. Cerebral, thoracic and abdominal arteries may be involved. Emergency and intensivist physicians, should be aware of the risk of occult traumatic vascular injury.

## Abbreviation

NF-1: neurofibromatosis type 1
